# Aerodigestive Foreign Bodies in Adult Ethiopian Patients: A Prospective Study at Tikur Anbessa Hospital, Ethiopia

**DOI:** 10.1155/2014/293603

**Published:** 2014-04-15

**Authors:** Abebe Bekele

**Affiliations:** Department of Surgery, School of Medicine, Addis Ababa University, Ethiopia

## Abstract

*Introduction*. Foreign bodies (FBs) in the aerodigestive tract are important causes of morbidity and mortality and pose diagnostic and therapeutic challenges. The best method of removal of an esophageal and tracheobronchial FB is endoscopic guided extraction. *Objective*. To present our experience of the removal of aerodigestive FBs in adult Ethiopian patients using rigid endoscopes. *Methods*. A hospital-based prospective study, at Tikur Anbessa Referral and Teaching Hospital, from January 2011 to December 2012 (over two years). *Results*. A total of 32 patients (18 males and 14 females) with a mean age of 28.0 ± 12.74 years were treated for FB ingestion and aspiration at Tikur Anbessa Hospital. The FBs were impacted at the esophagus in 18 (56.2%) patients, at the pharynx in 7 (21.8%), and at the air way in 7 (21.8%) patients. Pieces of bones were the commonest objects found in the esophagus (17/18 of the cases) and the pharynx (4/7), while fractured tracheostomy tubes and needles were frequently seen in the air way (3/7 cases each). The foreign bodies were visible in plain radiographs of 26 (81.2%) patients. Successful extraction of FBs was achieved by using Mc gill forceps in 11 cases, rigid esophagoscopes in 9 patients, and bronchoscopes in 4 cases. Four cases required open surgery to remove the foreign bodies. Two complications (one pneumothorax and one esophageal perforation) occurred. All patients were discharged cured. *Discussion and Recommendations*. Aerodigestive FBs are not so rare in the hospital and timely diagnosis and removal of accidentally ingested and aspirated foreign body should be performed so as to avoid the potentially lethal complications associated. Rigid esophagoscopy requires general anesthesia and is associated with its own complications, but our experience and outcome of its use are encouraging.

## 1. Introduction

Foreign bodies (FBs) in the aerodigestive tract are important causes of morbidity and mortality in the two extremes of life and pose diagnostic and therapeutic challenges [1]. The ingestion and aspiration of FBs occur most commonly in children's population, especially in their first six years of life [1–3]. However, they are not so uncommon in adults [4, 5]. Most FB ingestions in adults are related to eating, leading to either bone or meat bolus impaction, while poor dentition, inadequate chewing, and eating while being sedated can precipitate this problem [5, 6]. Food impaction may also indicate obstructive esophageal preexisting lesions such as esophageal (mucosal) ring, peptic or malignant esophageal stricture, or eosinophilic esophagitis [6, 7].

Adults account for only about 20% of the reported cases of aspirations [8]. The leading causes are associated with altered mental status, trauma with a decreased level of consciousness, and impaired airway reflexes, when airway protective mechanisms function inadequately or facial traumas. However, there is a distinct group of patients such as young Muslim ladies who frequently use Hijab pins who are being recognized and are at risk [8–11].

The best method of removal of an esophageal and tracheobronchial FB is endoscopic guided extraction [3–5]. However, the endoscopic method of choice has remained controversial. Over the past decade, the flexible fiberoptic esophagoscope has gained great popularity [2–4]. However, the rigid endoscope is equally effective in the hands of an experienced surgeon. Both rigid and flexible bronchoscopes can attain above 90–95% success rate [8], but there is no consensus as to which is better.

The most commonly used method in our hospital for removal of such FBs has been rigid endoscopy, mainly due to the lack of flexible scopes [12]. Hence, the purpose of this study is to present our experience of the removal of aerodigestive FBs in adult Ethiopians using rigid endoscopes under general anesthesia, with a review of the pertinent literature.

## 2. Methods

This is a prospective analysis of patients admitted for removal of FBs from the aerodigestive tract at Tikur Anbessa Hospital from January 2011 to December 2012. The hospital is the main teaching and referral hospital of Addis Ababa University, where patients with aspirated and swallowed FBs are mainly referred to and treated. Data collected in the study included age and sex of the patient, time elapsed before presenting to the hospital, type and location of the foreign bodies, diagnostic and treatments techniques utilized, and short-term follow-up of the patients. The FBs locations were recorded as pharyngeal, upper esophageal (between 15 and 28 cm from the incision teeth), middle esophageal (between 28 cm and 34 cm), distal esophageal (34 cm to the lower esophageal sphincter), tracheal, and main bronchial regions.

All procedures were performed after patients were admitted to the hospital and under general anesthesia. When FBs were visible in the pharynx or in the accessible segment of the upper esophagus, extraction was performed by Mc gill forceps. Rigid bronchoscopes and rigid esophagoscopes were utilized when the objects were deeper in the aerodigestive tract or when Mc gill forceps extraction was impossible. Esophagotomy and bronchotomy were also required in some cases (see Table 4). After each procedure, patients were observed in the hospital to see whether complications occurred or not. One follow-up visit one month after discharge was arranged for all patients. Data was collected using a structured questionnaire and analysis done by using EP-INFO-2002 statistical software.

## 3. Results

A total of 32 patients (18 males and 14 females) were treated in the hospital during the study period and included in the study. Their mean age was 28.0 ± 12.74 (range, 15–70) years. Twenty-one (65.4%) of patients were aged between 15 and 30. Nineteen (59.3%) patients presented to the hospital within 24 hours and 4 (12.5%) patients came after five days. One particular patient came after 2 months (see Table 1). The FBs were impacted at the esophagus in 18 (56.2%) patients (9 in the upper esophagus and 9 in the middle esophagus), at the pharynx in 7 (21.8%) patients, and at the air way in 7 (21.8%) patients (3 left main bronchi, 3 right main bronchi, and 1 trachea) (see Tables 2 and 3).

All patients with pharyngeal and esophageal foreign bodies presented with dysphagia and odynophagia, while one patient complained of additional severe left-sided neck pain. All patients with airway foreign bodies had cough and shortness of breath, one patient presented with severe upper airway obstruction, and one presented with recurrent respiratory tract infection.

Pieces of bones were the commonest objects found in the esophagus (17/18 of the cases) and the pharynx (4/7), while fractured tracheostomy tubes and needles were frequently seen in the air way (3/7 cases each). The tracheostomy tubes were permanently inserted for complicated thyroidectomy (1 patient), previous cut throat injury (1 patient), and unidentified indication (1 patient). Since all were not performed on in the study hospital, details of the patient were not available. Other impacted foreign bodies included Hijab pins, leech, and metal pieces in 1 (3.1%) patient each. Plain CXR was performed in all patients and foreign bodies were visible in 26 (81.2%). The six (18.8%) nonvisualized objects included 2/21 of the bone fragments, 2/2 of the wooden pieces, 1/3 of the broken plastic tracheostomy tubes, and 1/1 of the leech.

In all the 7 patients with the foreign body stuck in the pharynx and 4/9 of the proximal esophageal foreign bodies, the objects were successfully removed with the help of Mc gill forceps and laryngoscopes. These include 8/21 of the bone pieces, 1/2 of the pieces of wood, and 1/1 of leech and piece of metal (see Tables 2 and 3). Rigid endoscopy was used in 14 patients with esophageal foreign bodies and successful foreign body removal was accomplished in 9 patients (4/9 upper esophagus and 5/9 midesophagus). Four were disimpacted and were found difficult to grasp and hence were pushed to the stomach (all midesophageal).

One patient with a proximal esophageal FB required esophagotomy and extraction of the object. This was a 33-year-old male who swallowed a bone fragment 8 days before presentation complaining of dysphagia, severe neck pain, and neck swelling. His neck X-ray revealed a big bony lesion in the cervical esophagus and endoscopy showed a sharp speculated big piece of bone stuck at the proximal esophagus, perforating it at 3 and 9 o'clock. Therefore, left lateral neck incision was done and there was collected pus which was drained, the foreign body extracted with difficulty and the esophageal lacerations were debrided and repaired over an NG tube. The neck incision was drained and a feeding gastrostomy was placed. The incision drained for about two weeks and the patient showed gradual but complete recovery within three weeks and was discharged cured. Follow-up after 3 and 6 months revealed a completely healthy patient with no complications (see Tables 2 and 3).

Two-thirds of main bronchial bodies in each side were successfully removed with a rigid bronchoscope, while thoracotomy and bronchotomy were required in two patients (one long needle on the left and one fractured tracheostomy tube on the right). There was one tracheal fractured piece of a tracheostomy tube in the trachea that required emergency tracheotomy.

There were two complications seen. One patient developed pneumothorax after the extraction of a sharp Hijab pin from the left main bronchus which required chest tube drainage for three days. One esophagoscopy to remove a midesophageal bone fragment that was stuck for two months was successful but was complicated by esophageal perforation. This was successfully treated with prolonged right-sided chest drainage and a gastrostomy tube feeding. None of the patients died. All patients were followed up for one month after discharge and there were no short-term complications seen.

## 4. Discussion

Endoscopy has been the mainstay of management of aerodigestive foreign bodies [3, 4, 10–17]. Both rigid and fiberoptic esophagoscopes reportedly have similar success and morbidity rates [14]. The literature recommends flexible endoscopy (esophagoscopy and bronchoscopy) as cost effective because it is performed on an outpatient basis without general anesthesia, but, when sharp, penetrating, or difficult foreign bodies are present, rigid esophagoscopy is required [3, 4]. However, endoscopy does pose its own risks of complications, including failure of the procedure, bleeding, bronchospasm, accidental extubation, postprocedure stridor, hypoxia, esophageal perforation, and mediastinitis [10, 11].

Rigid endoscopy has a larger lumen and allows removal of most objects under direct vision [13]. The endotracheal intubation also provides an adequate airway and minimizes the incidence of aspiration during the procedure. Weisberg and Refaely and Al-Qudah et al. have also recommended the use of the rigid endoscope as the instrument of choice for extracting foreign bodies from the esophagus [14, 15]. Our method of endoscopic extraction has been the rigid scope, primarily because this has been the traditional approach in the hospital and the availability of flexible scopes was not regular. However, as reported by other studies done in the country [4], timely diagnosis and removal of accidentally ingested foreign body by flexible endoscopes can be practiced in Ethiopia.

Plain radiography on two planes has been recommended as an initial screening method in patients suspected with foreign bodies [7, 8]; our diagnostic yield has been 81.2%. Other studies have reported a detection rate of 47–75% [5, 10]. The use of barium swallow is discouraged by some authors since it may impair subsequent endoscopic visualization and increase the patient's aspiration risk [6].

Upper airway FBs are not frequently occurring phenomena in adults. As reported by Ramos et al. of the 9781 bronchoscopies performed in one center, only 32 involved cases of bronchoaspiration of FBs [10]. One of the largest series published identified 65 adults with tracheobronchial FB aspiration over a period of 12 years [18]. In our series FBs in the respiratory tract only represented 21% of the cases. Risk factors in adults include older age, abuse of sedative medications, neurological disorders (vascular dementia, Parkinson's), mental retardation, trauma with loss of consciousness, dental manipulations and procedures, alcoholism, and medical procedures, such as those resulting from cleaning or manipulating tracheostomy cannulas [9–12]. However, in contrast to these reports, our patients were found to be significantly younger and the only identifiable risk factor was the presence of tracheostomy tubes. The other types of FBs aspirated were sharp pins (needles and Hijab pins). Aspiration of such objects may occur when the pins are held between the lips and the individual forcefully inhales or coughs.

Air way FBs are potentially life threatening conditions that need to be addressed as soon as they are diagnosed or suspected. In most published series, the FBs tended to localize in the right bronchial tree [10, 18]. This right-side predominance can be explained by the vertical nature of the right main bronchus, its larger diameter, the greater air flow through it, and the localization of the carina to the left of the midline of the trachea [10]. In our series, 42.8% of the FBs were situated on each main bronchus and 14. 4% were in the trachea.

In our series, we could remove the FB by bronchoscopy in 3 (42.8%) of the cases and open surgical techniques (bronchotomy and tracheotomy) were required in 4 (57.2%). A high incidence of surgical treatment is also reported by other studies [10]. This fact could be explained by scarring and dense adhesion of the FB to the airway following recurrent infections, the special nature of some FBs that makes them difficult to grasp by forceps, and maybe our very low threshold for operative extraction when faced with difficult bronchoscopies. However, none of the patients were complicated with atelectasis or pneumonia.

A FB which failed to progress distally in the esophagus should be removed as soon as possible [1–5]. The rationale includes the following. Once an object is impacted in the esophagus the chance of spontaneous passage is small, edema from local trauma tends to grip the object more firmly making later manipulation increasingly difficult, and perforation of the esophagus is much more serious and dangerous than perforation of any other part of the gastrointestinal tract. Delay in presentation, diagnosis, or treatment will also result in complications [6–8]. This is supported by the fact that one of our patients who has swallowed a sharp piece of bone and presented after 5 days with esophageal perforation and cervical abscess collection. Some authors recommend that when cervical esophageal perforation is diagnosed after 48 hours, the treatment of preference is lateral neck incision, abscess drainage, foreign body extraction, limited attempt of esophageal repair, and prolonged drainage of the incision with nutritional support [16]. We have followed this protocol and our patient showed a complete recovery.

Fracture of tracheostomy tube with subsequent migration into the tracheobronchial tree is very uncommon and carries the potentially fatal risk of respiratory obstruction causes of FBs in the airway [17]. However, from our 7 patients with airway FBs, three had aspirated the fractured distal limb of a plastic tube. In one patient, the tube was lodged at the trachea and caused sudden potentially lethal airway obstruction. As these tubes can induce inflammation in the mucosal wall and cause fibrous adhesion, endoscopic extraction was not possible in 2 patients. Therefore, patients with tracheostomy must receive adequate information about this complication, in addition to their regular follow-up care.

In conclusion, aerodigestive FBs are not so rare in the hospital and timely diagnosis and removal of accidentally ingested and inhaled foreign body should be performed so as to avoid the potentially lethal complications associated. Rigid esophagoscopy requires general anesthesia and is associated with its own complications, but our experience and outcome of its use are encouraging.

## Figures and Tables

**Table 1:** Sociodemographic features of patients who underwent foreign body extraction at Tikur Anbessa Hospital, 2011-2012.

Characteristics	Frequency (*N* = 32)	Percentage (100%)
Age in years		
15–20	11	34.3
21–30	10	31.1
31–40	8	25
41–50	1	3.1
51–60	1	3.1
61–70	1	3.1
Sex		
Male	18	56.3
Female	14	43.7
Time between incident and presentation		
<6 hours	12	37.5
6–24 hours	7	21.8
24–48 hours	5	15.6
>48 hours	8	25

**Table 2:** Patters and location of foreign bodies extracted from patients who underwent endoscopic foreign body extraction in Addis Ababa, 2011-2012.

Foreign bodies extracted	Trachea	Right main bronchus	Left main bronchus	Pharynx	Upper esophagus	Middle esophagus	Frequency (*N* = 32)	Percentage %
Bone	—	—	—	4	8	9	21	65.6
Hijab pins	—	1	—	—	—	—	1	3.1
Leech	—	—	—	1	—	—	1	3.1
Metal pieces	—	—	—	1	—	—	1	3.1
Needle	—	—	3	—	—	—	3	9.4
Tracheostomy tube	1	2	—	—	—	—	3	9.4
Piece of wood	—	—	—	1	1	—	2	6.2

Total	1	3	3	7	9	9	32	

**Table 3:** Types of foreign bodies and techniques of extraction in Addis Ababa, 2011-2012.

Foreign body	Esophagoscopy	Esophagotomy	Mc gill forceps	Dislodged and pushed	Tracheotomy	Bronchotomy	Bronchoscopy	Total
Bone	8	1	8	4				21
Hijab pins							1	1
Leech			1					1
Metal pieces			1					1
Needle						1	2	3
Tracheostomy tube					1	1	1	3
Piece of wood	1		1					2

Total	9	1	11	4	1	2	4	32

**Table 4:** Site of impaction and the techniques of foreign body extraction utilized in Addis Ababa, 2011-2012.

Techniques of extraction	Trachea	Right main bronchus	Left main bronchus	Pharynx	Upper esophagus	Middle esophagus	Total
Bronchotomy	0	1	1	0	0	0	2
Bronchoscopy	0	2	2	0	0	0	4
Esophagoscopy	0	0	0	0	3	6	9
Esophagotomy	0	0	0	0	1	0	1
Mc gill forceps	0	0	0	7	4	0	11
Pushed down	0	0	0	0	1	3	4
Tracheotomy	1	0	0	0	0	0	1

Total	1	3	3	7	9	9	32
